# Lymphatic Valve Dysfunction in Western Diet-Fed Mice: New Insights Into Obesity-Induced Lymphedema

**DOI:** 10.3389/fphar.2022.823266

**Published:** 2022-03-04

**Authors:** Jorge A. Castorena-Gonzalez

**Affiliations:** Department of Pharmacology, School of Medicine, Tulane University, New Orleans, LA, United States

**Keywords:** lymphatic vessel, lymphatic valve, back-leak, obesity, lymphedema, obesity-induced lymphedema, plasminogen activator inhibitor-1, western diet

## Abstract

A two-way connection between obesity and lymphatic dysfunction has now been established. Clinical studies have demonstrated that obesity significantly increases the risk for developing secondary lymphedema. Using animal-models, obesity and metabolic syndrome have been linked to different aspects of lymphatic structural abnormalities and lymphatic dysfunction, including impaired contractility, impaired flow-mediated responses, impaired fluid transport, as well as increased permeability, and abnormal dendritic cell migration among others. Dysfunction of lymphatic valves is a main form of lymphatic dysfunction, known to result in severe edematous phenotypes; however, the extent of lymphatic valve deficiency in secondary lymphedema, including obesity-induced lymphedema, remains unknown. Therefore, the aims of the present study were 1) to determine whether western diet-induced obesity results in lymphatic valve dysfunction, and 2) to determine whether lymphatic valve dysfunction in western diet-induced obesity results from the diet itself, or as a consequence of the metabolic alterations induced by the diet. First, we quantitatively assessed and compared valve function in isolated popliteal and mesenteric collecting lymphatic vessels from control and western diet-induced obese C57BL/6J (WT) mice. Feeding a western diet for 14 weeks induced obesity and elevated plasma glucose and cholesterol levels when compared to controls. The function of lymphatic valves in popliteal lymphatics was not affected by diet-induced obesity; however, significant back-leak of pressure was observed in mesenteric lymphatic valves. Dysfunctional, leaky valves from obese animals also required significantly higher adverse pressure to trigger valve closure. Importantly, when subjected to treatment with a western diet, globally deficient PAI-1 mice were significantly protected against metabolic dysfunction and displayed fully functional, competent mesenteric lymphatic valves. In conclusion, our findings show for the first time that, in association with the metabolic alterations induced by the western diet, lymphatic valve dysfunction can be a critical component of obesity-induced lymphedema.

## Introduction

Deficient transport of lymph by a dysfunctional lymphatic system can lead to abnormal accumulation of fluid in the interstitial spaces, known as lymphedema. Importantly, it is now recognized that obesity significantly increases the risk for developing secondary lymphedema. As a significant health problem, obesity-induced lymphedema continues to gain attention by researchers and physicians as the clinical evidence continues to grow in association with the current obesity epidemic. According to the World Health Organization, in 2016, over 650 million adults were obese, i.e., body mass index greater than or equal to 30 kg/m^2^ (World-Health-Organization et al., 2021). The undoubtful connection between obesity and lymphatic dysfunction has become particularly evident by the increasing number of cases of morbidly obese patients that present with severe cases of secondary lymphedema (Farshid and Weiss, 1998; Chopra et al., 2015). Clinical studies have shown that morbidly obese patients are at significantly higher risk of developing lower-extremity lymphedema, i.e., abnormal fluid drainage, as determined by lymphoscintigraphy (Greene et al., 2012). Furthermore, patients with a body mass index greater than ∼50 kg/m^2^ have over 200-times greater odds of developing highly severe cases of lymphedema, i.e., massive localized lymphedema (Maclellan et al., 2017).

It is plausible that lymphatic dysfunction is present, with different degrees of severity, in all obese patients irrespective of their body mass index; however, in most cases, lymphatic dysfunction and its potential contributing role to the development and/or worsening of other cardiovascular diseases remain subclinical, and therefore unappreciated.

In studies using animal-models, obesity has been connected to different aspects of lymphatic dysfunction. For instance, obese and type-2 diabetic mice displayed increased lymphatic vascular permeability (Scallan et al., 2015); in a rat model for metabolic-syndrome, the intrinsic contractility of mesenteric lymphatics was impaired (Zawieja et al., 2012) and thoracic ducts displayed blunted flow-mediated responses and reduced expression of nitric oxide synthase (Zawieja et al., 2016); and in wild-type mice, feeding of a high fat diet resulted in reduced contractile frequency and impaired response to mechanostimulation in collecting lymphatics *in vivo* (Blum et al., 2014), and impaired lymphatic fluid transport and impaired dendritic cell migration (Weitman et al., 2013). A more complete and in-depth discussion of the available studies focusing on the understanding of how lymphatic function is regulated/dysregulated in obesity has been presented in recent reviews (Kataru et al., 2020; Antoniak et al., 2021).

Dysfunction of lymphatic valves is one of the main forms of lymphatic dysfunction. Utilizing mice, genes including *Foxc2*, *Rasa1*, *Piezo1*, and *Gja1* have been shown to play critical roles in the correct development and maintenance of lymphatic valves, as mutations to these genes result in abnormally developed, deficient lymphatic valves (Sabine et al., 2015; Lapinski et al., 2017; Munger et al., 2017; Nonomura et al., 2018). In humans, mutations to these genes have been associated with primary lymphedema. Relevant to this study, the extent of lymphatic valve deficiency in secondary lymphedema, and in particular that induced by obesity, remains unknown.

Therefore, in the present study we hypothesized that western diet-induced obesity in mice results in lymphatic valve dysfunction. As an important biomarker for disease, upregulation of the Plasminogen Activator Inhibitor-1 (PAI-1) has been linked to metabolic syndrome, obesity, inflammation, tissue fibrosis, and cardiovascular disease among others. Globally deficient PAI-1 (PAI-1^−/−^) mice have previously been shown to be protected against the metabolic effects of a high fat diet (Wang et al., 2018). We investigated whether western diet fed PAI-1^−/−^ mice also display a similar protection from metabolic dysfunction, and subsequently prevents lymphatic valve dysfunction. To test our hypotheses, we quantitatively assessed and compared the valve function in isolated collecting lymphatic vessels from control and diet-induced obese C57BL/6J (WT) and PAI-1^−/−^ mice. Quantitative assessment of valve function, i.e., capability of preventing back-leak of pressure when challenged with an adverse pressure gradient, was completed using a method recently developed by us (Castorena-Gonzalez et al., 2020). This is the first study that reports on quantitative assessment of lymphatic valve function in an animal model of obesity, and the first to link obesity with lymphatic valve dysfunction.

## Materials and Methods

### Ethics Statement

All animal protocols and procedures were approved by the Institutional Animal Care and Use Committees of Tulane University and the University of Missouri. These were performed in accordance with the NIH Office of Laboratory Animal Welfare’s Public Health Service Policy on Humane Care and Use of Laboratory Animals and Guide for the Care and Use of Laboratory Animals.

### Animals

*C57BL/6J* (Cat.No. 000664) WT male mice were purchased at 4 weeks of age from The Jackson Laboratory—Bar Harbor, MA, United States (JAX). Male and female PAI-1^−/−^ breeder mice were purchased from JAX (Cat. No. 002507) and animals for experiments were obtained through in-house breeding. Prox1-CreER^T2^ mice were obtained from Taija Mäkinen, University of Uppsala, Sweden and XIMBIO—London, England, and subsequently crossed to Rosa26 ^mT/mG^ reporter mice (JAX, Cat. No. 007676). Male WT and PAI-1^−/−^ mice were randomly divided and housed in groups of 2-3 animals per cage. Mice were housed at a temperature of 22–25°C under a 12-h light/dark cycle. Mice had uninterrupted access to food and water. At 5 weeks of age, groups of animals were randomly selected to either continue to receive regular mouse chow as a control diet (CD, PicoLab RodentDiet 20) or to switch to a western diet (WD, ResearchDiets Western Diet Cat.No. D12079Bi) for a period of 14 weeks. Both CD and WD are 20% protein diets, carbohydrate and fat content and the number of calories that each of these three macronutrients provide are different. In the CD, protein, fat, and carbohydrates provide 25, 13, and 62% kcal, respectively; while in the WD, these provide 17, 40, and 43% kcal. At the end of the diet treatment, mice were anesthetized by means of intraperitoneal injection of Ketamine/Xylazine (0.1 ml/25 g) and placed on a heated pad for blood collection and tissue dissection. Adequate levels of anesthesia were ensured by continuous monitoring for indicators of pain and by assessing loss of pedal and pinna reflexes. Following dissection, animals were euthanized via overdose of Ketamine/Xylazine and subsequent cervical dislocation.

### Assessment of Glucose, Triglycerides, and Cholesterol Levels

With a mouse anesthetized and prior to tissue dissections, blood was collected via cardiac puncture using a 1 ml syringe and a 23G needle, both pre-coated with EDTA (Invitrogen UltraPure 0.5 M EDTA, pH 8.0 Cat. No.: 15575-020). Drawn blood (usually 500 µl) was transferred into EDTA pre-coated 1.5 ml centrifuge tubes. Additional EDTA was added to reach a final concentration of 5 mM. Once transferred, blood was gently hand-mixed and placed in ice. Blood samples were centrifuged at 1,500 × G and 4°C for 15 min using a pre-cooled Thermo Scientific Sorvall ST 8R centrifuge. After centrifugation, the supernatants were collected and transferred into fresh 0.5 ml microcentrifuge tubes and immediately snap frozen in liquid nitrogen. Plasma samples were then stored at −80°C. Glucose, triglycerides, HDL, and LDL/VLDL cholesterol concentrations were then assessed in these plasma samples using a glucose colorimetric detection kit (Invitrogen Cat. No.: EIAGLUC), a triglycerides assay kit (Bioassay Systems Cat. No.: ETGA200), and a cholesterol assay kit (Cell Biolabs Inc. Cat. No.: STA391), respectively.

### Vessel Isolation, Pressure Myography, and Data Acquisition

Popliteal and mesenteric lymphatic vessels were isolated as previously described (Scallan and Davis, 2013; Scallan et al., 2015; Castorena-Gonzalez et al., 2018; Zawieja et al., 2018). Briefly, for popliteal lymphatic vessels, starting with the mouse in the prone position laying on a heated tissue dissection/isolation pad, the superficial saphenous vein was exposed by making a proximal-to-distal incision along the calf area beginning at the ankle and the popliteal afferent lymphatic vessels that run along this major vein were isolated; and for mesenteric lymphatic vessels, starting with the mouse in supine position, the intestines were exteriorized and excised, then unfolded and pinned down onto a Sylgard-coated 100-mm petri dish containing Krebs buffer supplemented with bovine serum albumin (BSA, denoted as Krebs-BSA). This facilitated the localizing and dissection of lymphatic vessel tissues from the jejunal area. Then, each popliteal or mesenteric lymphatic tissue was pinned down onto the Sylgard-coated surface of a dissection chamber filled with Krebs-BSA where most of the adipose and connective tissues surrounding the vessel were micro-dissected. A partially clean vessel segment was then transferred to an observation chamber filled with Krebs-BSA solution. The vessel segment was cannulated and pressurized to 3 cmH_2_O, under no-flow conditions, using two glass micropipettes. Although the resistance of these pipettes was not determined (inside diameter was ∼40–50 µm), we used the same pair of inflow and outflow pipettes for all valve tests included in this study. Micropipettes were set up on pipette holders mounted on the MX10R micro-manipulators (Siskiyou Corporation) of our in-house designed and assembled observation pressure myography chamber. To ensure accurate diameter tracking, the cannulated vessel segment was further cleared of remaining connective and adipose tissue. Lymphatic segments contained a single valve. The pipette system with cannulated lymphatic vessel segment was transferred onto the XY-stage of an inverted microscope for observation and experimentation. Polyethylene tubing was attached to the back of each glass micropipette and then connected to a microfluidic flow control system (Elveflow OB1 MK3, Paris) with attached low-pressure transducers. Consistent with previous studies (Davis et al., 2011; Lapinski et al., 2017; Castorena-Gonzalez et al., 2018; Castorena-Gonzalez et al., 2020), to minimize longitudinal bowing and associated diameter-tracking artifacts at higher intraluminal pressures, input (P_input_) and output (P_output_) pressures were briefly set to 10 cmH_2_O at the beginning of every experiment, and the vessel segment was stretched axially to remove longitudinal slack. The vessel was then allowed to equilibrate at 37°C for 30 min in a Ca^2+^-free Krebs buffer while both input and output pressures were set to 3 cmH_2_O. Constant perfusion with Ca^2+^-free Krebs buffer was maintained using a peristaltic pump at a rate of 0.5 ml/min. Custom-written LabVIEW programs (National Instruments; Austin, TX) acquired real-time analog data and digital video (Davis et al., 2006). Videos were recorded in brightfield mode at 30 fps using a Basler acA2000-340 km camera.

### Lymphatic Valve Function: Measurement of Pressure Back-Leak and Assessment of Valve Gating

The competency and degree of pressure back-leak across a lymphatic valve challenged with an adverse pressure gradient were assessed, under Ca^2+^-free conditions, using a simplified method we recently developed (Castorena-Gonzalez et al., 2020). In brief, intraluminal pressures, i.e., P_input_ and P_output_, were set to 0.5 cmH_2_O, after diameter reached a steady level (usually 2-3 min), each isolated valve was then subjected to an increasing adverse pressure gradient by raising P_output_ from 0.5 to 10 cmH_2_O in a ramp-wise manner at a rate of 15 cmH_2_O/min, while P_input_ was maintained at 0.5 cmH_2_O, this protocol is referred to as back-leak test ramp. As output pressure increased, changes in outside diameter were tracked at the input side, i.e., lymphangion upstream from the valve. The basic principle behind these measurements is that a fully functional and competent lymphatic valve closes in the presence of an adverse pressure gradient, the overlapping, closed leaflets prevent back-leak of pressure, and therefore changes in diameter, into the protected upstream lymphangion. However, the defective seal formed by the leaflets of a dysfunctional valve may allow back-leak of pressure into the upstream lymphangion, resulting in changes in outside diameter even when pressure at the input pipette is maintained at 0.5 cmH_2_O. The magnitude of the changes in vessel diameter at the input side is directly associated with the degree of lymphatic valve dysfunction. To quantify the degree of pressure back-leak, the changes in outside diameter at the input side were then mapped against calibration pressure curves generated, for each lymphatic vessel segment, by simultaneously increasing P_input_ and P_out_ from 0.5 to 10 cmH_2_O. Alternating sets of back-leak test ramps and calibration curves were repeated 2-3 times and the resulting measurements/calculations of pressure back-leak were then averaged for each tested valve. Brightfield videos were recorded for each back-leak test ramp and calibration curve, and then automatically processed and analyzed to determine the degree of pressure back-leak using software tools developed in-house using Python. Consistent with our previous studies, to minimize hysteresis in these pressure-diameter curves, the vessel wall was unloaded by briefly setting intraluminal pressure to 0 cmH_2_O for about 10–20 s, prior to each pressure ramp (either calibration or back-leak test), and then pressure was set to 0.5 cmH_2_O and diameter was allowed to equilibrate as mentioned above.

Following the assessment of pressure back-leak, each individual single-valve lymphatic segment was subjected to a series of valve gating pressure ramps, which allowed to determine the adverse pressure gradient required to be present across a valve to drive it from an open to a closed position. Similar to back-leak pressure ramps, valve gating ramps consisted of increasing P_output_ starting at a baseline value that matched P_input_. Valve gating was assessed under 5 different baseline pressure conditions, i.e., 0.2, 0.5, 1, 2, and 3 cmH_2_O. The maximum tested adverse pressure gradient was 20 cmH_2_O. Each valve gating test was performed 3 times at each pressure condition, and the measured values were averaged.

### Solutions and Chemicals

During microdissection and cannulation of lymphatic segments, a Krebs-BSA buffer was utilized. It contained: 146.9 mM NaCl, 4.7 mM KCl, 2 mM CaCl_2_·2H_2_O, 1.2 mM MgSO_4_, 1.2 mM NaH_2_PO_4_·H_2_O, 3 mM NaHCO_3_, 1.5 mM Na-HEPES, 5 mM d-glucose, and 0.5% BSA (pH = 7.4). For valve testing, cannulated and pressurized vessel segments were perfused with a Ca^2+^-free Krebs buffer (where 3 mM EGTA replaced calcium). All chemicals were obtained from Sigma-Aldrich, United States (St. Louis, MO, United States).

### Cross-Sectional Distensibility Calculation

Using Python-based software tools developed in-house and the brightfield videos recorded of calibration pressure curves (i.e., P_input_ and P_output_ increased simultaneously in a ramp-wise manner in the range of 0.5–10 cmH_2_O), we automatically tracked both internal and external diameters of a lymphatic vessel segment as a function of intraluminal pressure. Then, for a given *k-th* pressure, the cross-sectional distensibility (D_CS_) was calculated as previously described (Foote et al., 2016):

$$D_{\mathrm{CS},k}=\frac{\mathrm{CS}A_{k}-\mathrm{CS}A_{i}}{\left[\mathrm{CS}A_{i}\times \left(P_{k}-P_{i}\right)\right]}$$ (1)

where CSA is the lymphatic vessel cross-sectional area, a function of the inner diameter and wall thickness, and the *i-th* index refers to the initial, baseline state (i.e., minimum tested pressure equals 0.5 cmH_2_O of intraluminal pressure).

### Lymphatic Vessel RNA Isolation and Quantification Using Droplet Digital PCR

Expression of *Serpine1*, encoding PAI-1, was quantitatively assessed using droplet digital PCR (ddPCR). Mesenteric lymphatic vessels were isolated and cleaned as described in *Vessel Isolation, Pressure Myography, and Data Acquisition*. After dissection, intact lymphatic vessel segments were rinsed by transferring them into 1.5 ml centrifuge tubes containing 1 ml of sterile PBS and then spun down by centrifugation at 900 × G and 4°C for 2 min. PBS was then removed, leaving only the vessels segments pelleted to the bottom of the tube, and finally, tubes were transferred to liquid nitrogen for freezing and subsequently stored at −80°C until tissues were processed. Lymphatic vessels from mice fed either a control or a western diet (*N* = 4 for each group) were included in these experiments. Due to the small size of these lymphatic segments and overall limited number of available vessels after other experimental protocols had been completed, each ddPCR sample contained pooled segments from two animals. Total RNA was isolated using the Arcturus PicoPure RNA Isolation Kit (ThermoFisher Scientific, Waltham, MA Cat. No.: KIT0214). ddPCR blind experiments were performed in collaboration with the *Molecular Core Facility of the Tulane Hypertension and Renal Center of Excellence* using a Bio-Rad ddPCR system as previously described (Yanofsky et al., 2021). All reagents, primers, and probes were purchased for their use with the One-Step RT-ddPCR system (Bio-Rad). A mouse *Serpine1* PrimePCR ddPCR Gene Expression Probe Assay was utilized and purchased from Bio-Rad (Cat.No.: 10031252, Assay ID: dMmuCPE5124194). PAI-1 mRNA levels were normalized by *β*-actin levels.

### Real Time RT-PCR on FACS-Purified Lymphatic Cells

Inguinal axillary lymphatic vessels were isolated from tamoxifen-induced Prox1-CreER^T2^;Rosa26 ^mT/mG^ mice. For Cre-recombinase induction, mice were fed with a tamoxifen-containing diet for 7 days (ENVIGO TD.130855). Vessels were isolated 2 weeks post-induction. Isolated and cleaned lymphatic vessel segments were enzymatically digested and cells were dispersed as previously described (Davis et al., 2020). Lymphatic endothelial cells (LECs) expressing eGFP (i.e., mG^+^) as well as non-recombined tdTomato-expressing cells (i.e., mT^+^) were sorted by means of Fluorescence-Activated Cell Sorting (FACS) with a Beckman-Coulter MoFlox XDP system at the *Cell and Immunology Core Facility* of the University of Missouri. Total RNA was extracted from FACS-sorted mG^+^ (i.e., LECs) or mT^+^ cells using the Arcturus PicoPure RNA Isolation Kit (ThermoFisher Scientific, Waltham, MA, United States, Cat. No.: KIT0214) with on-column DNase I treatment (Qiagen, Valencia, CA, United States) according to manufacturer’s instructions. RNA was eluted with 25 μl nuclease-free water. Purified RNA was transcribed into cDNA using the High-Capacity cDNA Reverse Transcription Kit (ThermoFisher Scientific, Waltham, MA, United States). Real-time PCR was performed on cDNAs prepared from each sample using 2x SYBR Green qPCR Master Mix (Bimake, Houston, TX, United States) with primers listed in Supplemental Table S1. Approximately 50–60 sorted cells were used per reaction and data collection was carried out using a Bio-Rad CFX 96 Real-Time Detection System (software version Bio-Rad CFX Manager 3.1; Bio-Rad, Hercules, CA, United States). Real-time PCR protocols were performed as follows: preheating at 95°C for 3 min, 40 cycles of two-step cycling of denaturation at 95°C for 15 s and annealing/extension steps of 45 s at 60°C. A melting curve was tested after each run to confirm the specificity of the amplified products and no template conditions were included to test for contamination of assay reagents. For analysis, the results were expressed as a ratio between the target gene and a reference gene (i.e., *Gapdh*).

### Statistical Analyses

The number N refers to the number of animals utilized in this study, while *n* refers to the total number of experiments (e.g., single-valve lymphatic segments tested independently). In most cases, more than one lymphatic segment from the same mouse was successfully studied. Depending on the data sets being compared and the different variable-interactions being assessed, different parametric or non-parametric, paired, or unpaired statistical tests with appropriate multiple-comparison post hoc corrections were performed. A detailed description of the statistical tests utilized for each data set is included in each figure caption. Normality of the data distributions was assessed using the Shapiro-Wilk and Kolmogorov-Smirnov tests. Results are reported as mean ± SEM with significance set at *p* < 0.05. All statistical analyzes were performed using GraphPad Prism 9.

## Results

### Feeding of a Western Diet Induced Obesity, High Blood Glucose, and High Cholesterol Levels in Mice

**FIGURE 1:**
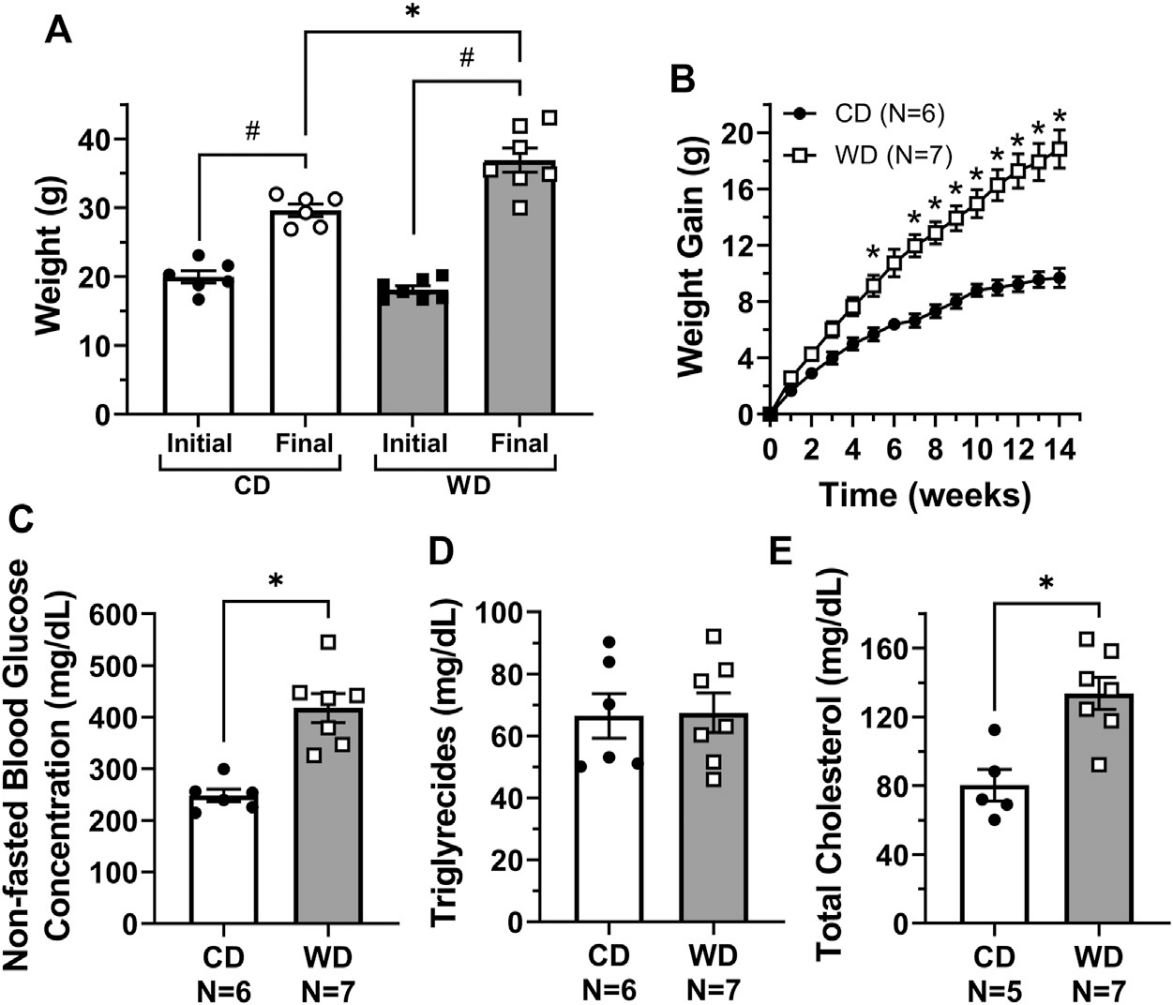
Feeding of a western diet for 14 weeks induced obesity and increased plasma glucose and cholesterol levels. **(A)** Initial and final body weight for control diet (CD) and western diet (WD) groups; **(B)** overall gain in body weight per week over the 14-week treatment period; and **(C–E)** non-fasted plasma glucose, triglycerides, and total cholesterol concentration (in mg/dL) for CD and WD groups, respectively. In panel E, cholesterol levels could not be measured in one sample from the CD group due to limited sample. Data is presented as mean ± SEM. Statistically significant differences were evaluated using t tests for data in panels A, and C–E, and two-way ANOVA with Šídák post hoc test for data in panel B. ^#^ *p* < 0.05 for initial vs. final weights, *p < 0.05 for CD vs. WD.

Consistent with many other studies, feeding of a western diet (WD, *N* = 7) for 14 weeks resulted in significant gain of body weight, i.e., 18.8 ± 1.4 g, vs. mice fed a control diet (CD, *N* = 6), which gained 9.7 ± 0.7 g (Figures 1A,B). Based on the weekly food consumption (in g) per cage, we calculated the mean food intake per mouse. Weekly food intake in control animals was higher (25.5 ± 1.8 g per mouse) when compared to animals fed a WD (20.1 ± 0.7 g per mouse). Utilizing the Atwater general factors for metabolizable energy (Buchholz and Schoeller, 2004), the calculated weekly metabolizable energy per mouse was 86.8 ± 6.0 and 93.5 ± 3.8 kcal for CD and WD groups respectively. In the fed state, glucose, triglycerides, and cholesterol (HDL, VLDL/LDL, and total) levels were assessed from plasma samples. Non-fasted blood glucose concentration was significantly higher in diet-induced obese mice when compared to controls (418 ± 28.0 and 248.0 ± 12.2 mg/dl respectively, Figure 1C). Triglycerides were similar in both groups (Figure 1D). Total cholesterol was significantly higher in WD-fed animals (134.0 ± 9.4 mg/dl) vs. controls (80.4 ± 9.2 mg/dl) (Figure 1E). Importantly, VLDL/LDL levels were not different between diet groups (24.4 ± 5.2 mg/dl vs. 28.2 ± 6.9 mg/dl for CD and WD, respectively); however, the significant increase in total cholesterol was associated with higher HDL in the WD-fed group (105.0 ± 12.6 mg/dl) vs. the CD-fed group (56.0 ± 12.0 mg/dl).

### Diet-Induced Obesity and Associated Metabolic Alterations Result in Mesenteric, but not Popliteal, Lymphatic Valve Dysfunction

**FIGURE 2:**
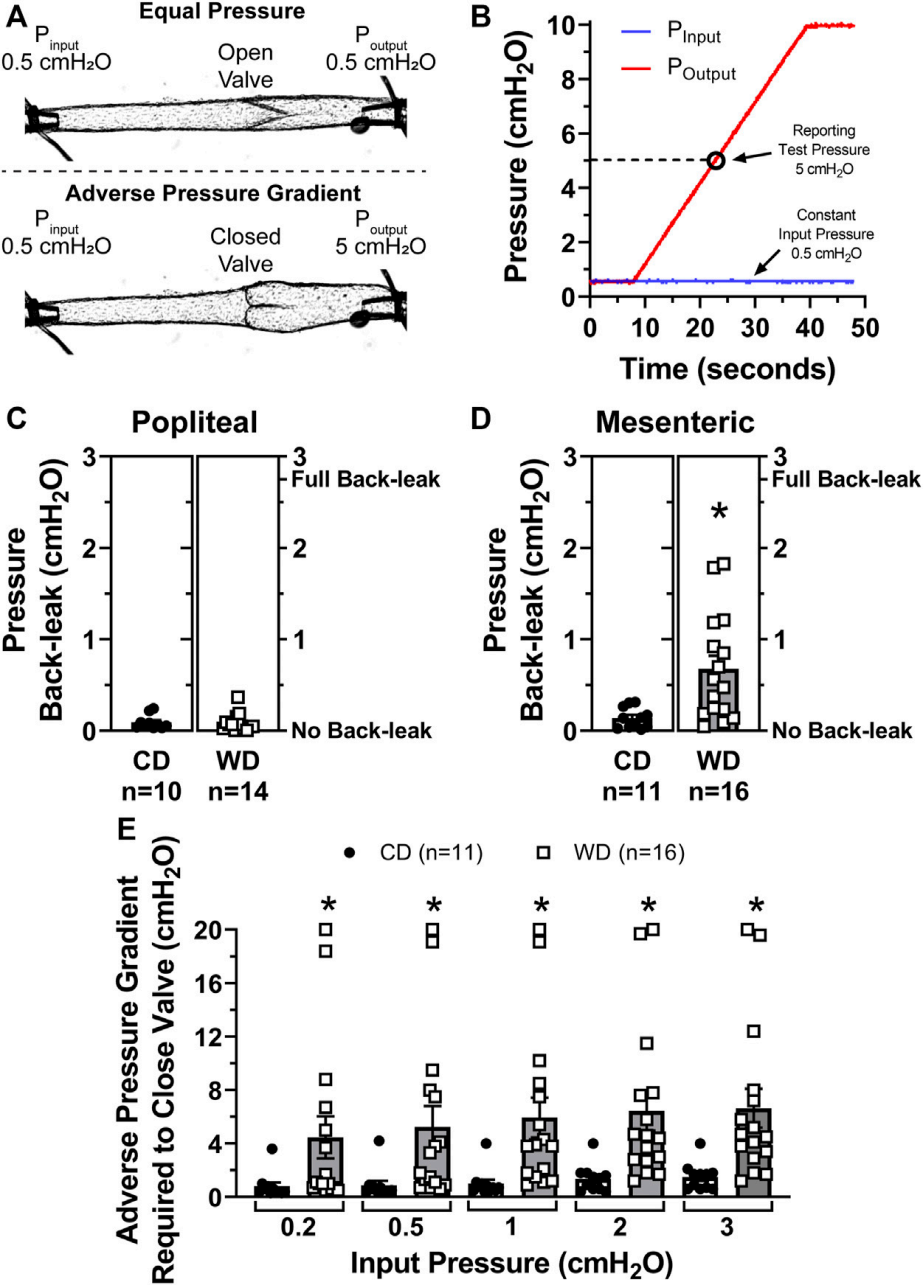
Diet-induced obesity induced valve dysfunction in mesenteric but not popliteal lymphatic vessels. **(A)** Diagram of a representative valve test displaying the initial state, i.e., P_input_ = P_output_ = 0.5 cmH_2_O (valve open, top), and during valve challenge, i.e., P_input_ = 0.5 and P_output_ = 5 cmH_2_O (valve closed, bottom); **(B)** representative traces for P_input_ (blue) and P_output_ (red) pressures during a valve test. Results are reported when P_output_ = 5 cmH_2_O (open circle); **(C,D)** pressure back-leak measurements comparing CD and WD groups in popliteal and mesenteric lymphatics respectively. Each data point represents each individually tested valve. Multiple tests, i.e., 2-3, were performed on each valve, each data point represents the mean of all these tests; and **(E)** the adverse pressure gradient required to drive each specific valve onto a closed position when P_input_ was set and maintained at 5 different intraluminal baseline pressure levels (i.e., 0.2, 0.5, 1, 2, and 3 cmH_2_O). Data is presented as mean ± SEM. Datasets included in this figure were not normally distributed, therefore, statistical differences were evaluated using non-parametric *t* tests. *p < 0.05 for CD vs. WD.

We recently developed a method for the assessment of lymphatic valve function (Castorena-Gonzalez et al., 2020) that allows us to determine the degree of pressure back-leak through isolated lymphatic valves when challenged with an adverse pressure gradient. Single-valve popliteal afferent and mesenteric lymphatic vessel segments were isolated from control and diet-induced obese WT mice. We tested the function of these isolated valves and determined their capability to effectively prevent back-flow when subjected to an adverse pressure gradient (Figure 2A). To accomplish this, we measured pressure back-leak as a function of output pressure, P_output_ = 0.5 to 10 cmH_2_O, while input pressure (P_input_) was maintained constant at 0.5 cmH_2_O. Consistent with our previous study, results are here reported at P_output_ = 5 cmH_2_O (Figures 2A,B). The function of lymphatic valves in popliteal afferent lymphatic vessels was not affected in diet-induced obesity, i.e., no significant back-leak was observed in either CD (*n* = 10) or WD (*n* = 14) groups, indicative of fully competent valves (Figure 2C). Importantly, diet-induced obesity resulted in significant back-leak of pressure in mesenteric lymphatic valves (Figure 2D).

### Dysfunctional Mesenteric Lymphatic Valves in Western Diet Fed Mice Require a Higher Adverse Pressure Gradient to Close

When an adverse pressure gradient is present across a lymphatic valve, its leaflets are driven to a closed position (Figures 2A,B). Based on previous studies, we know that: 1) valves close when an adverse pressure gradient reaches a threshold pressure, referred to as valve gating pressure gradient; 2) this valve gating pressure gradient depends on the level of pressure present upstream from the valve (i.e., input side); and 3) structural characteristics of the valve, including (but not limited to) leaflet length and vessel diameter upstream and downstream from the valve, are important determinants for valve closure and the degree of seal created when valves close (i.e., degree of leaflet overlap). Further testing on mesenteric lymphatic valves revealed that dysfunctional, i.e., leaky valves, from obese animals also required a significantly higher adverse pressure gradient to trigger valve closure (Figure 2E). This was assessed at different levels of upstream/input pressure (i.e., 0.2, 0.5, 1, 2, and 3 cmH_2_O). For instance, when P_input_ = 0.5 cmH_2_O, the same pressure condition under which back-leak measurements were assessed, the adverse pressure gradient required to close a given valve was 0.9 ± 0.3 cmH_2_O for valves in the control diet group, vs. 5.5 ± 1.6 cmH_2_O for valves isolated from diet-induced obese animals.

### Lymphatic Valve Dysfunction in Western Diet Fed Animals is Associated With Increased Vessel Diameter to Leaflet Length Ratios

**FIGURE 3:**
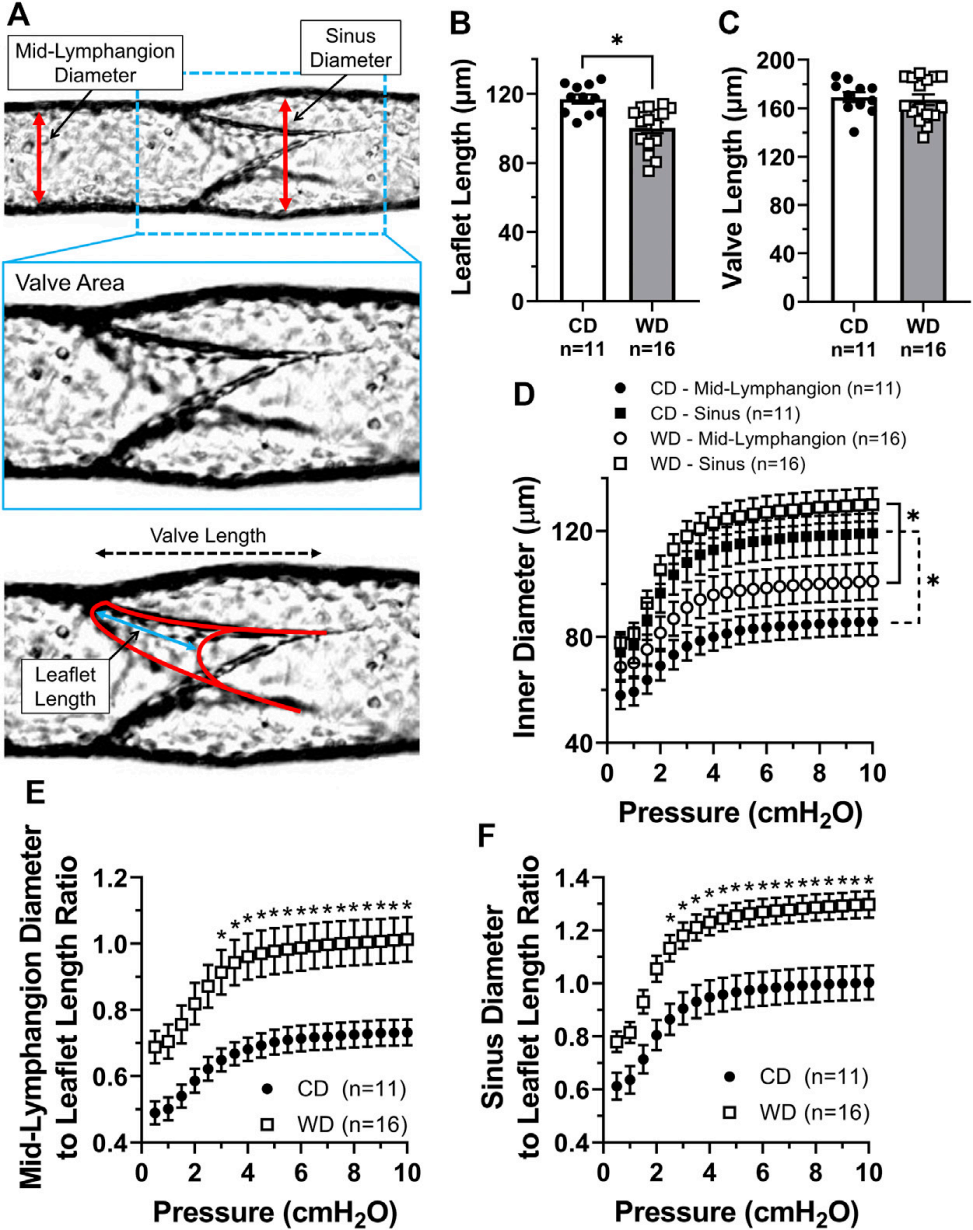
Increased lymphatic vessel diameter to leaflet length ratios in western diet fed mice. **(A)** Diagram displaying the different parameters measured to assess morphological changes in lymphatic vessels and valves induced by obesity; **(B)** leaflet length; **(C)** valve length; **(D)** mean inner diameter, measured at mid-lymphangion and sinus areas, as a function of intraluminal pressure; **(E)** ratio between the mid-lymphangion vessel diameter and leaflet length as a function of pressure; and **(F)** ratio between the sinus diameter and leaflet length as a function of pressure. Data is presented as mean ± SEM. Statistically significant differences were evaluated using parametric t tests for data in panels B and C; two-way ANOVA with Tukey post hoc for data in panel D; and two-way ANOVA with Šídák post hoc test for data in panels E and F. *p < 0.05 for CD vs. WD, or other groups as designated.

To determine if obesity-induced dysfunction of mesenteric lymphatic valves involved any structural modifications, we quantified some of the main morphological parameters (Sabine et al., 2018) characterizing these single-valve lymphatic segments, i.e., leaflet length, valve length, and vessel diameter upstream from the valve (mid-lymphangion) and at the sinus area, Figure 3A. Utilizing brightfield recordings, leaflet and valve length manual measurements were performed under P_input_ = P_output_ = 3 cmH_2_O, while automated tracking/measurement of inner and outer diameters was performed as a function of intraluminal pressure using a Python-based tracking program developed in-house. Measurement of valve leaflet length showed that mesenteric valve leaflets in vessels from obese animals were ∼17 µm shorter than those from lean controls (117 ± 3 vs. 100 ± 3 µm respectively, Figure 3B). Overall length of mesenteric valves was not affected by western diet (Figure 3C). Mesenteric lymphatic vessels from obese animals displayed non-statistically different, enlarged diameters at both mid-lymphangion and sinus areas. Signs of early outward remodeling were particularly evident in the mid-lymphangion areas (Figure 3D). Importantly, the modest, but significant shortening of valve leaflets, and the enlargement of lymphatic vessels in western diet fed mice resulted in significantly increased vessel diameter to leaflet length ratios (Figures 3E,F).

### Cross-Sectional Distensibility is Differentially Increased at Lymphatic Valve Sinus Areas and Further Enhanced in Obesity

**FIGURE 4:**
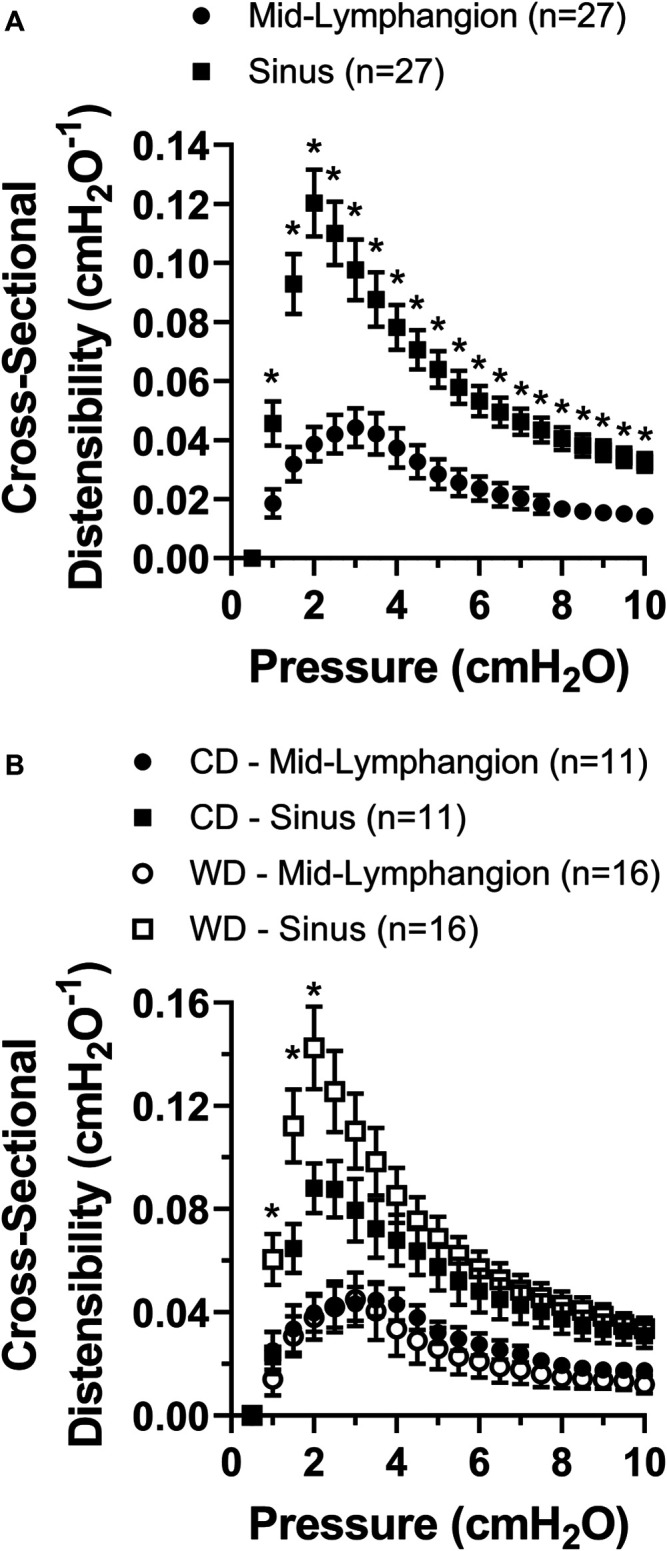
Cross-sectional distensibility is differentially increased in the sinus areas of mesenteric lymphatic vessels and it is increased in obesity. **(A)** Lymphatic cross-sectional distensibility as a function of intraluminal pressure measured at mid-lymphangion and sinus areas; and **(B)** lymphatic cross-sectional distensibility as a function of intraluminal pressure measured at mid-lymphangion and sinus areas for specific diet groups, i.e., CD and WD. Data is presented as mean ± SEM. Statistically significant differences were evaluated using two-way ANOVA with Tukey and Šídák post hoc. In panel A, *p < 0.05 indicates a significant difference was found between mid-lymphangion and sinus areas irrespective of the diet; and in panel B, *p < 0.05 indicates significant differences at sinus areas between CD and WD.

We assessed diet-induced changes in lymphatic vascular elasticity by comparing the cross-sectional distensibility of mesenteric lymphatic vessels from control vs. western diet fed mice. Using the automatically tracked inner and outer diameters from brightfield videos of lymphatic vessels subjected to a range of increasing pressures, we calculated the cross-sectional distensibility at sinuses and mid-lymphangion areas as a function of intraluminal pressure. Irrespective of the diet treatment, the cross-sectional distensibility of mesenteric lymphatic vessels was significantly higher at the sinus area compared to that measured at the mid-lymphangion area (Figure 4A). Importantly, the increased distensibility at sinus areas was further enhanced by diet-induced obesity in the low to mid-pressure range, i.e., ≤2 cmH_2_O (Figure 4B).

### Global PAI-1 Deficiency Ameliorates Metabolic Dysfunction, Preventing Lymphatic Valve Dysfunction in Mice Fed a Western Diet

**FIGURE 5:**
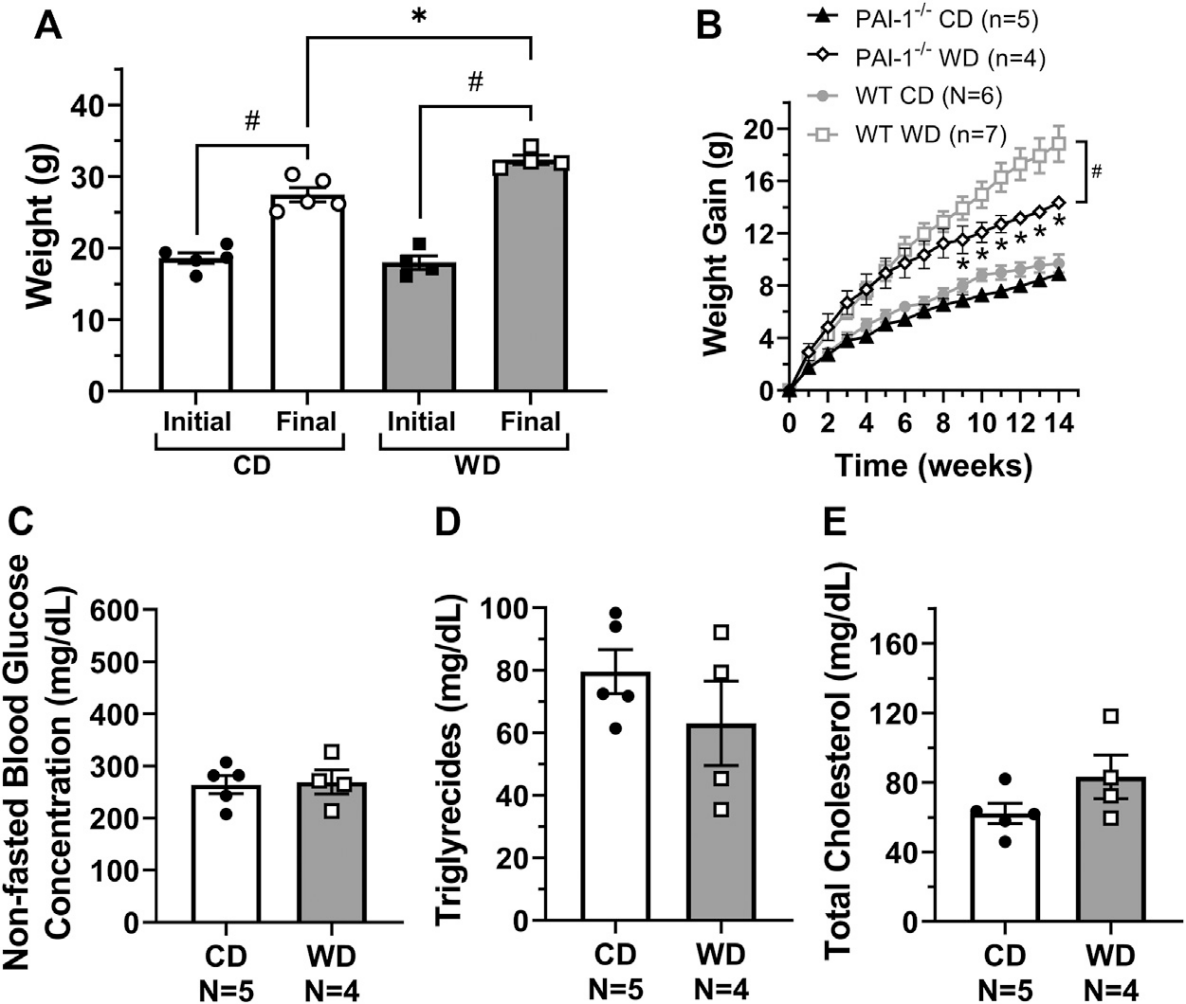
Global PAI-1 deficient mice are partially protected against metabolic dysfunction. For PAI-1^−/−^ mice, **(A)** initial and final body weight control diet (CD) and western diet (WD) groups; **(B)** overall gain in body weight per week over the 14-week treatment period (WT data from Figure 1B is also shown in gray); and **(C–E)** non-fasted plasma glucose, triglycerides, and total cholesterol concentration (in mg/dL) for CD and WD groups respectively. Data is presented as mean ± SEM. Statistically significant differences were evaluated using t tests for data in panels A, and C–E, and two-way ANOVA with Šídák post hoc test for data in panel B. In panel A, ^#^ *p* < 0.05 for initial vs. final weights; and in panel B, *p < 0.05 for CD vs. WD and ^#^ *p* < 0.05 for PAI-1^−/−^ WD vs. WT WD.

**FIGURE 6:**
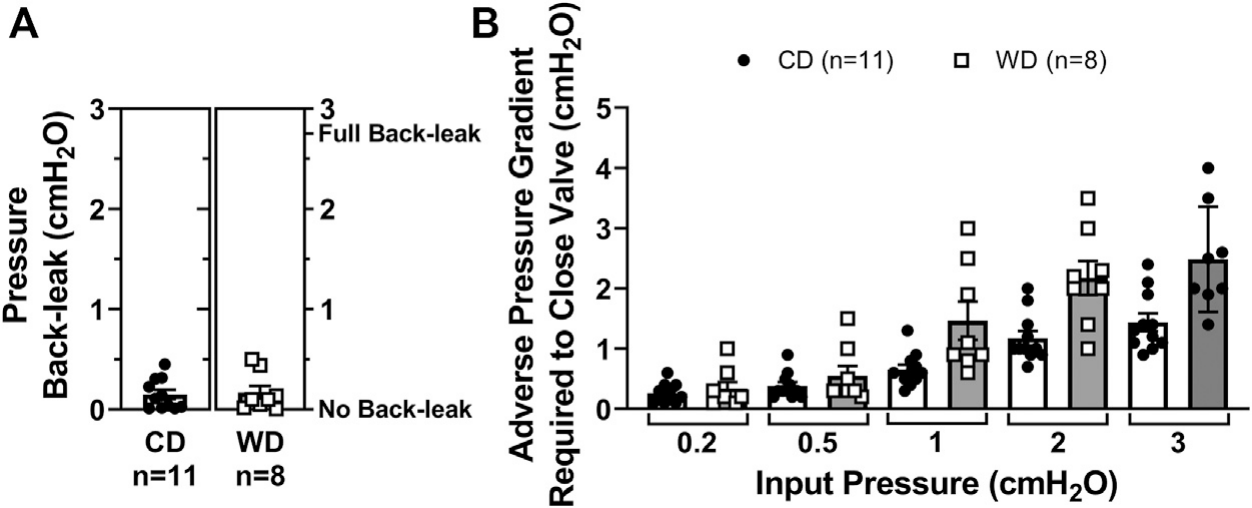
Improved metabolic function in western diet-fed PAI-1−/− mice prevents mesenteric lymphatic valve dysfunction. **(A)** Pressure back-leak measurements in mesenteric lymphatic valves from PAI-1^−/−^ mice fed a CD or a WD respectively. Each data point represents an individually tested valve. Multiple tests were performed, and resulting values were averaged for each valve; and **(B)** the adverse pressure gradient required to drive each specific valve onto a closed position when P_input_ was set and maintained at 5 different intraluminal baseline pressure levels (i.e., 0.2, 0.5, 1, 2, and 3 cmH_2_O). Data is presented as mean ± SEM. Datasets included in this figure were not normally distributed, therefore, statistical differences were evaluated using non-parametric *t* tests. No significant differences were found.

To determine if lymphatic dysfunction in western diet-induced obesity results from exposure to the components of the diet itself or in association with the metabolic effects induced by it, we fed male PAI-1^−/−^ mice with either a CD (*N* = 5) or a WD (*N* = 4) for 14 weeks. Globally deficient PAI-1 mice were partially protected against obesity; while PAI-1^−/−^ mice fed a WD gained significantly more body-weight than those in the control group, the degree of body-weight gain was also significantly less than WT mice fed a WD (Figures 5A,B). In addition, in contrast to WT mice, global deletion of PAI-1 completely prevented the significant increase in glucose and cholesterol levels observed after treatment with a western diet (Figures 5C–E). Importantly, competent mesenteric lymphatic valve function was observed in both control and WD-fed PAI-1^−/−^ mice, i.e., no significant back-leak of pressure was measured (Figure 6A). Furthermore, mesenteric lymphatic valves in both diet groups required similar adverse pressure gradients to drive their leaflets onto the closed position (Figure 6B). These levels of valve gating pressure were also comparable, i.e., non-statistically different, to those observed in mesenteric lymphatic valves from WT mice fed a control diet (Figure 6B vs. Figure 2E).

### PAI-1 is Expressed in the Lymphatic Vascular Wall and Upregulated in Diet-Induced Obese Mice

**FIGURE 7:**
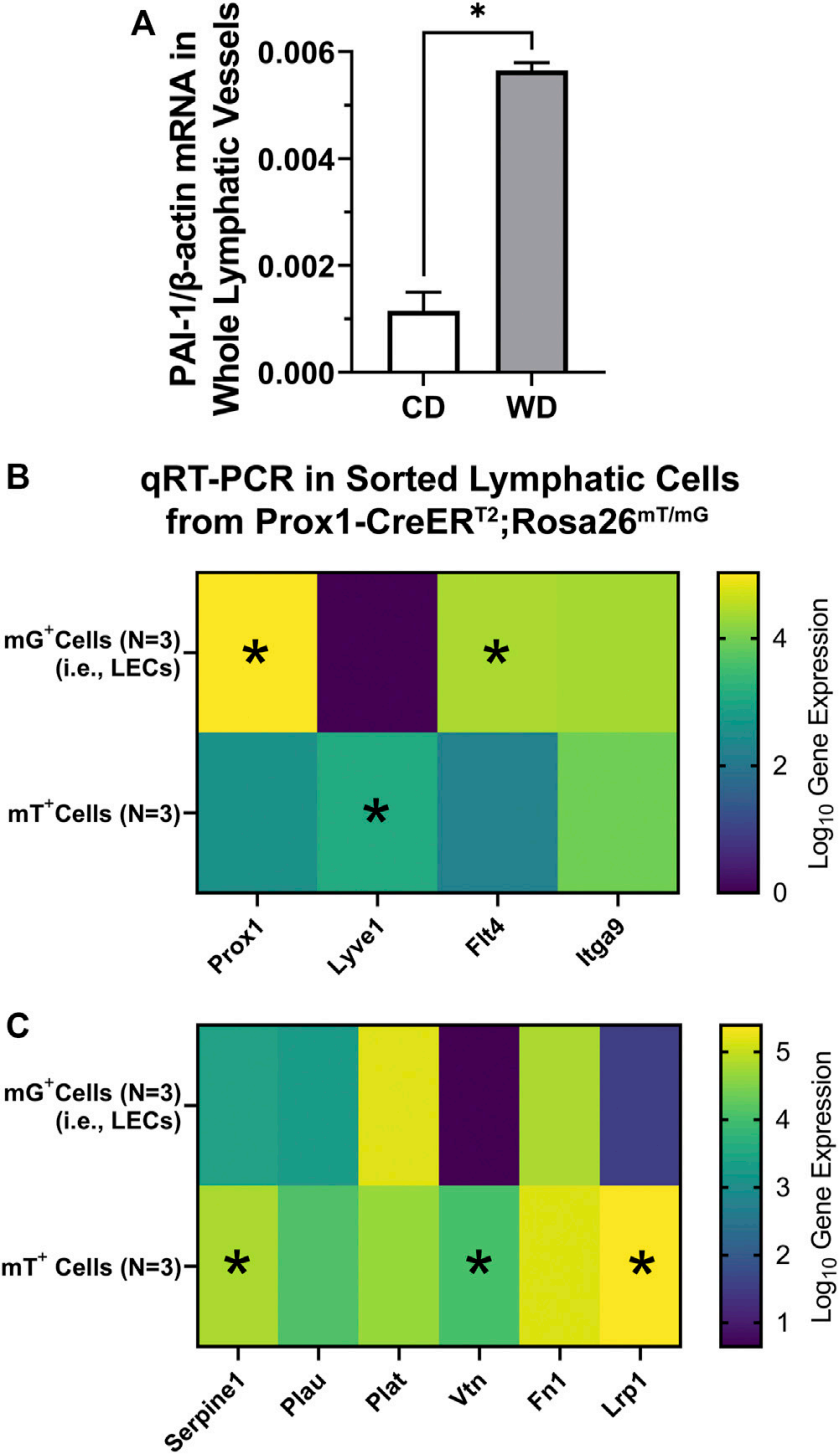
PAI-1 is expressed throughout the lymphatic wall and upregulated in diet-induced obesity. **(A)** PAI-1 mRNA expression (normalized by *β*-actin) assessed using ddPCR in whole mesenteric lymphatic vessels from control diet (CD) and western diet (WD) fed WT mice; **(B, C)** gene expression for various LEC-associated and PAI-1 associated genes using qRT-PCR on sorted mG^+^ (i.e., LECs) and mT^+^ cells from Prox1-CreER^T2^; Rosa26 ^mT/mG^ reporter mice. Data is presented as mean ± SEM. Statistically significant differences were evaluated using parametric *t* tests. *p < 0.05 for mG^+^ vs. mT^+^ cells.

As abnormal circulating and tissue-specific levels of PAI-1 are known to be present in disease, including obesity, we assessed whether PAI-1 is expressed in the lymphatic vasculature and whether its expression is altered in western diet-induced obesity. We quantified mRNA expression of *Serpine1* (gene encoding PAI-1) in whole mesenteric lymphatic vessels from control and diet-induced obese WT mice (4 animals per group) using ddPCR. *Serpine1* mRNA was detected in the wall of mesenteric lymphatics, and more importantly, its expression was significantly upregulated (>4-fold increase) in western diet fed mice (Figure 7A). To determine whether PAI-1 is expressed in lymphatic endothelial cells (LECs) and/or in other cell types within the lymphatic wall including lymphatic muscle cells (LMCs), we assessed the expression of *Serpine1* using qRT-PCR in FACS sorted mG^+^ (green) and mT^+^ (red) cells from enzymatically digested lymphatic vessels from tamoxifen-treated Prox1-CreER^T2^;Rosa26 ^mT/mG^ reporter mice (*N* = 3). Here, mG^+^ cells constitute a purified population of LECs, while the population of mT^+^ cells include a broad range of cell types including LMCs and even some potentially unrecombined LECs among others. We first characterized the expression of the LEC-associated genes *Prox1*, *Lyve1*, *Flt4*, and *Itga9* (Figure 7B). Significantly higher gene expression was observed for *Prox1* and *Flt4* in LECs (i.e., mG^+^ cells), while expression of *Itga9* was similar in both populations. Consistent with previous studies showing that *Lyve1* is downregulated in mature collecting lymphatic vessels (Makinen et al., 2005), expression of *Lyve1* was not detected in our purified samples of LECs. Interestingly, significant expression of *Lyve1* was observed in mT^+^ cells, indicating that other cell types in the lymphatic wall are *Lyve1* ^+^ (Figure 7B). We then assessed the expression of *Serpine1*, as well as the expression of genes that encode other proteins known to interact with PAI-1, i.e., *Plau* (uPA), *Plat* (tPA), *Vtn* (vitronectin), *Fn1* (fibronectin), and *Lrp1* (LDL receptor related protein 1). *Serpine1* was found in both populations of cells, i.e., LECs (mG^+^) and mT^+^ cells, indicating that PAI-1 is likely expressed, at the protein level, in various cell types throughout the lymphatic wall. Significantly higher expression of *Serpine1*, *Vtn*, and *Lrp1* was observed in mT^+^ cells (Figure 7C).

## Discussion

In the present study, utilizing a diet-induced obesity model in mice and a methodology recently developed by us for the quantitative assessment of valve function (Castorena-Gonzalez et al., 2020), we have demonstrated that western diet-induced obese mice display lymphatic valve dysfunction, i.e., leaky valves, in association with metabolic alterations including significant gain in body-weight, high blood glucose, and high cholesterol levels. Valve dysfunction in obese mice was observed in mesenteric lymphatics; however, lymphatic valves in peripheral popliteal lymphatics remained competent. This could be associated with the regional differences in the microenvironment that surrounds these vessels, e.g., the degree of inflammation associated with differential distribution of adipose tissue (Chusyd et al., 2016). Further testing on mesenteric lymphatic valves showed that dysfunctional valves from diet-induced obese animals also required abnormally higher adverse pressure gradients to trigger the closure of these deficient valves. Importantly, using a servo-null micro-pressure system, it was recently shown that in mesenteric collecting vessels from wild-type mice, the observed physiological pressures were <5 cmH_2_O (Czepielewski et al., 2021). Here, ∼1/3 of the valves in the obese WT mice group required pressure gradients exceeding 5 cmH_2_O to switch from an open to a closed position, suggesting that these valves are likely never closing under physiological conditions. Depending on the proportion of deficient valves and its severity, lymphatic valve dysfunction, i.e., severe chronic backflow, can cause fluid to back up along the lymphatic networks, increasing transmural pressure, and subsequently lead to vessel enlargement and increase in permeability, compromising fluid transport.

Assessment of structural and mechanical properties of the mesenteric lymphatic vessels from diet-induced obese WT animals did not reveal any robust structural or mechanical abnormalities, only modest changes were observed. In our present study, vessel enlargement did not reach statistical significance; however, previous studies have demonstrated lymphatic dysfunction associated with enlargement of the lymphatic vasculature in response to diet-induced obesity. For instance, collecting lymphatic vessels in the lower limb in mice fed a high-fat diet displayed enlarged diameters (Blum et al., 2014). The differences between the traditional high-fat diet and the western diet could explain the different phenotypes observed. Although higher in cholesterol content, the western diet provides 20% less kcal from fat when compared to the high-fat diet (i.e., 40% vs. 60% kcal respectively). Here, western diet fed WT animals displayed significantly increased levels of glucose and total cholesterol. Although these levels were assessed in non-fasted animals, the abnormally high glucose levels suggest that these mice were likely insulin resistant, which was not assessed. Insulin resistance (Cao et al., 2021) and high cholesterol could be important contributing factors to the dysfunction of the lymphatic vasculature in western diet fed mice, including valve dysfunction. The significant role of high cholesterol in lymphatic dysfunction was previously underscored by a study showing that hypercholesterolemic (ApoE^-/-^) mice displayed numerous structural and functional lymphatic abnormalities, including enlargement of initial lymphatic networks, decreased LMC coverage in collecting vessels, increased permeability, and impaired fluid transport (Lim et al., 2009).

Our results showed that, irrespective of the diet group, lymphatic vessels display differential cross-sectional distensibility between sinus and mid-lymphangion areas. Feeding of a western diet induced modest but significant changes in this mechanical parameter in the low-mid range of pressures (i.e., ≤2 cmH_2_O) but only at sinus areas, which have previously been shown to display significantly reduced coverage with LMCs (Zawieja et al., 2018). This raises an important question about obesity-driven alteration of LMC-coverage and its potential role in regulating valve function. Numerous previous studies have shown that vascular distensibility is an important factor/determinant of vascular, i.e., arterial/arteriolar, dysfunction in association with obesity, diabetes, and hypertension (Reneman and Hoeks, 1995; Van Bortel et al., 1995; Tentolouris et al., 2003; Bender et al., 2015; Foote et al., 2016; Ljunggren et al., 2016). Future studies from our lab, and others, could evaluate cross-sectional distensibility as a potential biomechanical indicator and determinant of lymphatic valve gating and valve function, adding to previously reported parameters (Davis et al., 2011; Lapinski et al., 2017).

Another biomechanical factor that likely has an important role in determining valve gating and valve function, one that has not been assessed due to experimental difficulty and technological limitations, is valve leaflet stiffness. This is particularly important regarding obesity-induced lymphedema as in the blood circulation, where vascular stiffening is known to be associated with obesity and is considered a significant predictor for the development of cardiovascular diseases. Our group is currently exploring novel experimental and analytical approaches to assess lymphatic valve stiffness *ex-vivo* in isolated lymphatic vessels.

Cooperatively, our observations and those reported in previous studies by others suggest that it is the collection of morphological and mechanical changes, in association with the various metabolic alterations in diet-induced obesity, that result in significant lymphatic dysfunction, including valve dysfunction. Therapeutic strategies for obesity-induced lymphedema likely require a multi-approach plan including the use of novel pharmacological aids, surgical and/or nutritional intervention for weight-loss, and exercise among others. Evidence against the success of single-approach strategies was previously provided by a clinical study that showed that massive weight-loss following sleeve gastrectomy (i.e., bariatric surgery) was not sufficient to reverse the obesity-induced lymphedema in morbid obesity (Greene et al., 2015). This suggests that the structural and functional modifications to the lymphatic vasculature induced by the chronic and highly inflammatory state associated with obesity and metabolic syndrome may not be reversible by the targeting of inflammation and/or weight-loss alone.

In conclusion, our findings show for the first time that lymphatic valve dysfunction, in association with the metabolic alterations induced by the western diet, can be a critical component of obesity-induced lymphedema. It is important to note that, in our present study, rescue of lymphatic valve function in western diet fed PAI-1^−/−^ mice is most likely a result of the overall improved systemic health, i.e., metabolic function, and prevented/lessened chronic inflammation. However, clinically significant unexplored areas of research, which are underscored by our results showing significant upregulation of *Serpine1* (PAI-1) in the lymphatic vasculature of western diet fed WT mice, are: 1) to determine the role of PAI-1 in regulating lymphatic function, including valve development, maintenance, and function; and 2) as one of the main functions of the lymphatic system is that of lipid absorption, determine whether PAI-1 plays a critical lymphatic-specific role (e.g., in lacteals and/or mesenteric lymphatics) in the regulation of metabolic function and overall systemic health. Our lab is currently seeking answers to these important questions, in the context of obesity and metabolic syndrome, in PAI-1^fx/fx^ mice crossed to *Lyve1*, *Prox1*, or *Myh11* Cre-lines.

## Data Availability

The original contributions presented in the study are included in the article/Supplementary Material, further inquiries can be directed to the corresponding author.
