# Supplementary figures and images for: Lymphatic Valve Dysfunction in Western Diet-Fed Mice: New Insights Into Obesity-Induced Lymphedema
Source: Front Pharmacol. 2022 Mar 4;13:823266. doi: 10.3389/fphar.2022.823266 (PMC8931217; doi:10.3389/fphar.2022.823266)

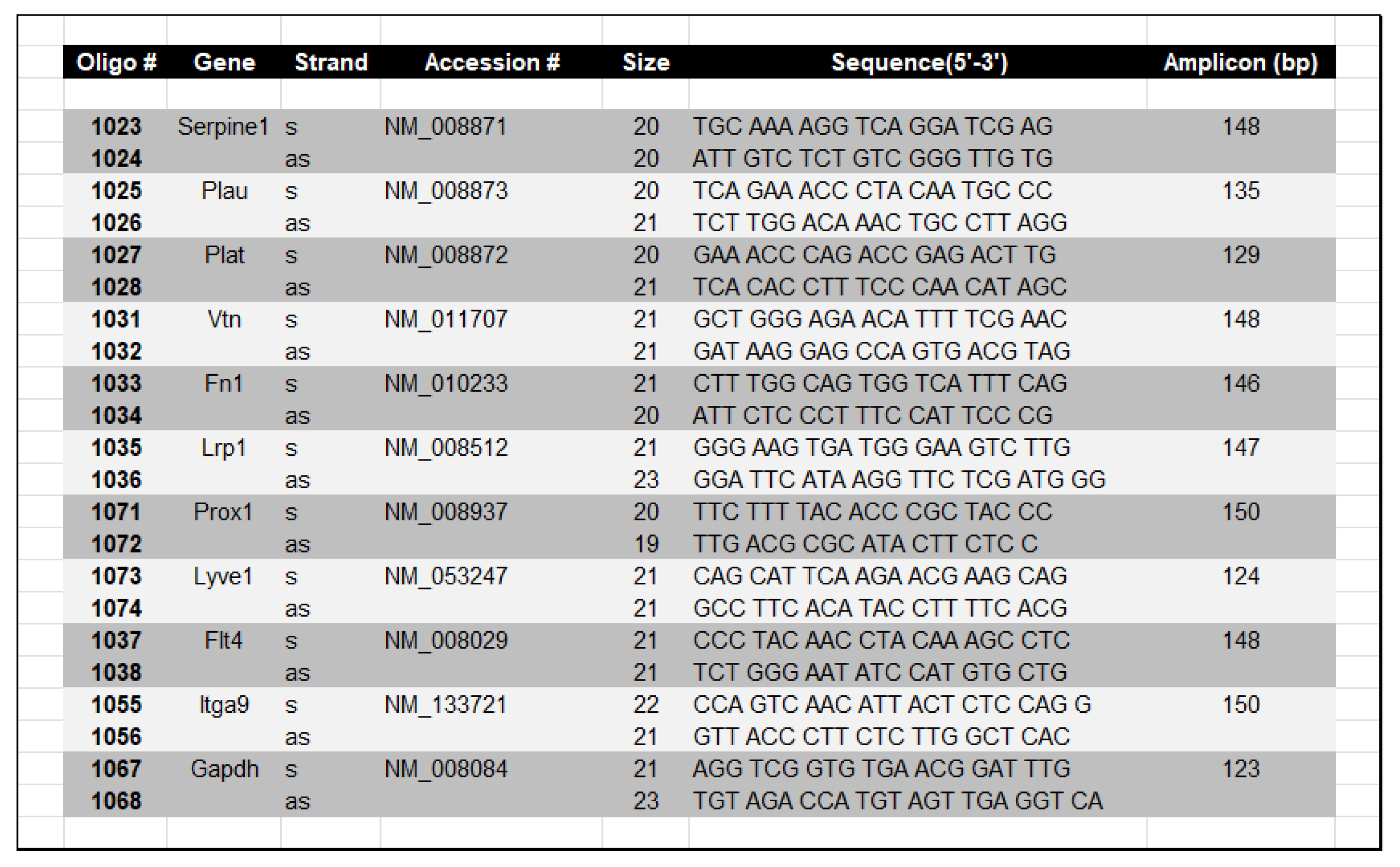

Supplement: Supplementary file 1 [file Image1.TIF]
